# A new model for diabetes-focused capacity building – lessons from Sri Lanka

**DOI:** 10.1186/s40842-018-0074-3

**Published:** 2018-12-12

**Authors:** Anjan K. Saha, Naresh Gunaratnam, Rashmi Patil, Monica Choo, Devika P. Bagchi, Ekta Jhaveri, Jennifer Wyckoff, Ganeika Bahu, Ulysses Balis, Paul Clyde, William H. Herman

**Affiliations:** 1 University of Michigan Medical School, Ann Arbor, MI USA; 2 The Grace Girls’ Home, Trincomalee, Sri Lanka; 3 The William Davidson Institute, University of Michigan, Ann Arbor, MI USA; 4 Department of Internal Medicine, Division of Metabolism, Endocrinology, and Diabetes, University of Michigan, Ann Arbor, MI USA; 5 Trincomalee General Hospital, Trincomalee, Sri Lanka; 6 Department of Pathology, Division of Informatics, University of Michigan, Ann Arbor, MI USA; 7 Michigan Diabetes Research Center, University of Michigan, Ann Arbor, MI USA

**Keywords:** Diabetes, Sri Lanka, Medical assistants, Capacity building

## Abstract

Sri Lanka is experiencing a rapid increase in the number of people with diabetes mellitus (DM) due to population growth and aging. Physician shortages, outdated technology, and insufficient health education have contributed to the difficulties associated with managing the burden of disease. New models of chronic disease management are needed to address the increasing prevalence of DM.

Medical students, business students, and faculty members from the University of Michigan partnered with the Grace Girls’ Home, Trincomalee General Hospital, and Selvanayakapuram Central Hospital to identify and train diabetes-focused medical assistants (MAs) to collect and enter patient data and educate patients about their disease. Return visits to these MAs were encouraged so that patient progress and disease progression could be tracked longitudinally. Data entry was conducted through a cloud-based mechanism, facilitating patient management and descriptive characterization of the population. We implemented this pilot program in June 2016 in coordination with Trincomalee General Hospital and Selvanayakapuram Central Hospital. Over a 12-month period, 93 patients were systematically assessed by the medical assistants. All patients received education and were provided materials after the visit to better inform them about the importance of controlling their disease. Fifteen percent (14/93) of patients returned for follow-up consultation.

Trained MAs have the potential to provide support to physicians working in congested health systems in low-resource settings. Public investment in training programs for MAs and greater acceptance by physicians and patients will be essential for handling the growing burden associated with chronic illnesses like DM. Trained MAs may also play a role in improved patient education and awareness regarding diabetes self-management.

## Background

By 2050, the world population ≥ 60 years of age is projected to be 1.5 times larger than the population < 10 years of age [1]. Noncommunicable diseases associated with aging represent an increasing public health challenge [2]. Globally, the top 10 causes of death in 2015 were dominated by noncommunicable conditions [3]. Of particular concern is the emergence of diabetes mellitus (DM) as the sixth most deadly health-related condition. The increased global morbidity and mortality associated with DM can be attributed to its rapid rise in prevalence in low- and middle-income countries (LMICs) [4]. Sri Lanka, an island nation just south of India, provides a microcosmic representation of the global challenges associated with DM in LMICs, from its increasing prevalence due to a growing and aging population to the challenges associated with managing its increased burden.

The prevalence of diabetes in Sri Lanka is increasing rapidly. In 2017, there were 1.2 million adults with diabetes reported in the nation [5]. The overall prevalence of DM in Sri Lanka for adults between the ages of 20 and 79 was 8.6%, with rates as high as 16.5% in urban areas [6]. Long-term diabetes prevention requires complementary dietary and lifestyle adjustments [7]. Successful diabetes management requires interventions including diet, physical activity, smoking cessation, pharmacologic management of glycemia, blood pressure, and lipids, and early detection and treatment of diabetic microvascular and neuropathic complications to prevent their devastating late sequelae, including blindness, kidney failure, and amputation [8]. Yet our global health management strategy, specifically in LMICs like Sri Lanka, is poorly equipped to accommodate the increased burden presented by chronic illnesses like DM. The severe shortage of medical professionals, especially licensed physicians, results in an increasing demand for care that is difficult to meet [9]. Most medical practices in the developing world do not make use of the latest developments in technology, such as modern electronic health records (EHRs) that provide easier access to clinical data, streamlined clinical workflows, and improved support for clinical-decision making and patient safety [10, 11]. Very few patients receive enough health education to fully understand how to care for their conditions [12].

Strategies to mitigate these systemic barriers might facilitate more efficient management of increasingly prevalent non-communicable diseases such as DM. A well-studied and validated method to address these systemic barriers involves expanding the number of ancillary providers [13]. Medical assistants (MAs) who perform routine administrative and clinical tasks to facilitate and reinforce physician care and physician extenders or advanced practice providers (physician assistants, nurse practitioners, and clinical pharmacists) who provide care under the supervision of a physician are playing increasingly important roles in care delivery [14–17]. Within diabetes care, certified diabetes educators (CDEs) are specifically trained to educate, support, and advocate for people affected by diabetes. Many CDEs are dually certified as pharmacists, expanding the level of care they can provide to their patients [18, 19]. While MAs and physician extenders have proven benefits to patient outcomes, these occupations are not well studied or publicly recognized in many LMICs, including Sri Lanka [13]. Moreover, prescribing medications in many LMICs is a responsibility held solely by physicians [20].

In this paper, we present an example of a cross-continental, multidisciplinary pilot study involving ancillary care providers, implemented for assessing and managing DM in Trincomalee, Sri Lanka. Medical students, business students, and faculty members from the University of Michigan partnered with the Grace Girls’ Home, Trincomalee General Hospital, and Selvanayakapuram Central Hospital to train diabetes-focused MAs to collect and enter patient data electronically in order to facilitate management and to educate patients about their disease. This paper details the strategy and results from initial implementation of a MA-driven program.

### Approach

The endeavor began as a joint effort between local physicians in Sri Lanka, the University of Michigan Medical School, and the University of Michigan Ross School of Business. The goal was to demonstrate that underutilized local human resources could be leveraged to build capacity to provide real-time data on a variety of diabetes directed metrics: i) the demographics of the patient population seen by the medical assistants; ii) the clinical profile of diabetes within the cohort; and iii) the treatment strategies being employed by patients. Our method for achieving this goal centered around training local personnel seeking employment in the health-care sector, with a desired outcome of improving care efficiency and quality while expanding the availability of services for people with both type 1 and type 2 diabetes.

**Fig. 1:**
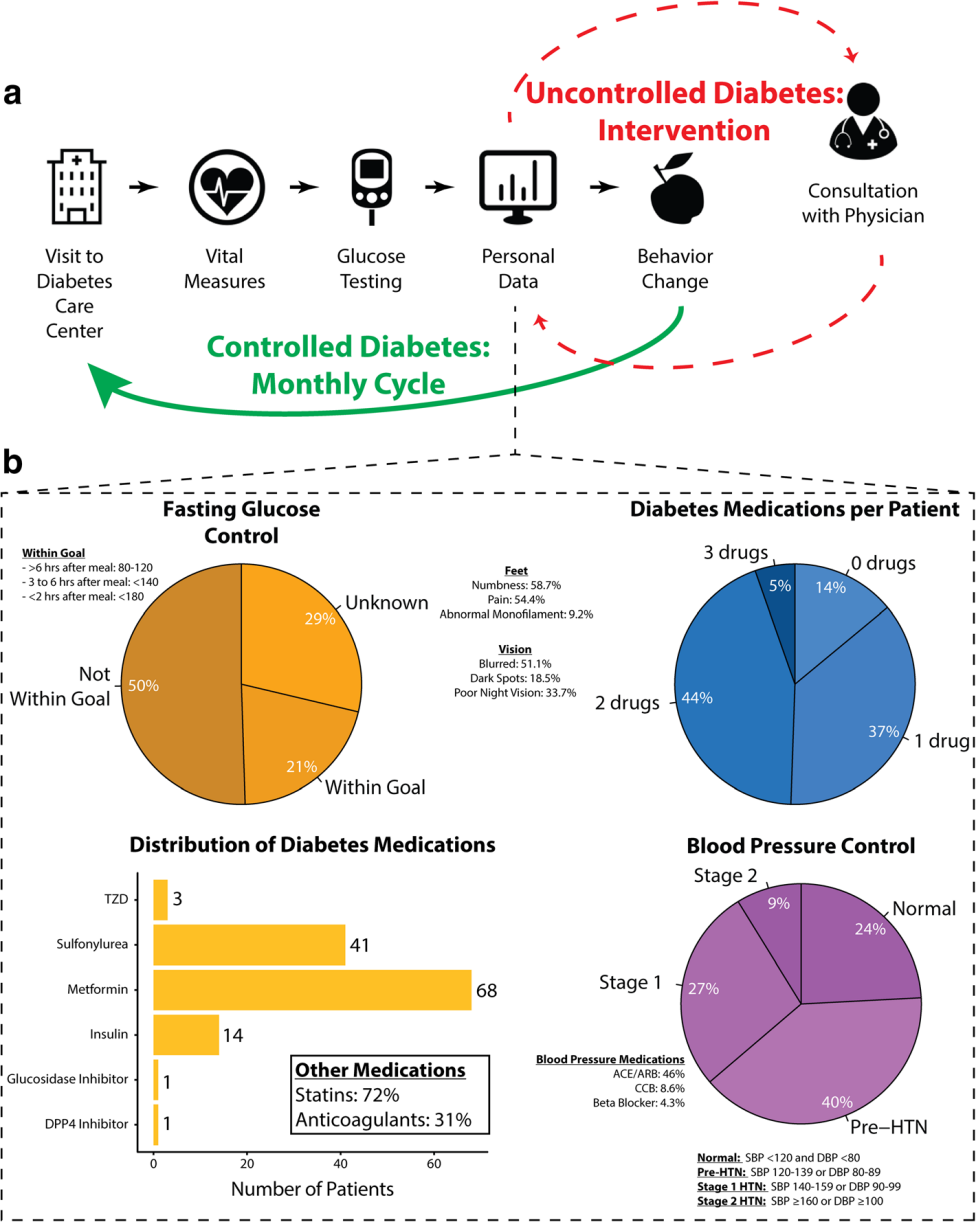
Diabetes Clinic Work Flow. A depiction of the care delivery model. **a** Patients were met at either Trincomalee General Hospital or Selvanayakapuram Central Hospital by the medical assistants who have a specialized skillset for managing diabetes. They enter clinical and objective data into a lightweight EHR/web application (MiDiET) that is built to run a back-end triage algorithm to evaluate disease control. **b** Representative data demonstrating that our model has the capacity to produce clinically and academically useful data

Interviews conducted with health ministry officials and local physicians confirmed that the management of diabetes suffers from systemic congestion, defined by shortages in health care personnel and high patient volumes. Developing measures to relieve this congestion was identified as a top priority. The utilization of the diabetes-focused MAs was proposed as an approach to mitigate this congestion, by expanding the pool of ancillary health professionals to streamline care. MAs were favored over other physician extenders given the legal restrictions on who is able to prescribe medications (physicians). The MA-driven model was designed by business students affiliated with the William Davidson Institute at the University of Michigan, an independent non-profit organization dedicated to providing private-sector solutions to LMICs. The model was initially focused on providing employment opportunities in Trincomalee’s health sector to underprivileged young adults. Medical students, general practitioners, and endocrinologists subsequently adopted the model, determined the infrastructural and medical constraints, and transformed the program from one that solely provided employment opportunities to one that also has the capacity to capture relevant biometrics that can inform and optimize diabetes management (Fig. 1a).

The team of endocrinologists determined which variables the MAs should capture when engaging patients. These variables include assessment of glycemic control, blood pressure, medication regimens, date of diagnosis, and pertinent history and physical exam findings. These variables were entered by the MAs into a web-based lightweight EHR, termed the Michigan Diabetes Evaluation Tool (MiDiET) developed by the informatics team. The application uses web responsive architecture to ensure compatibility across most widely utilized digital devices, from handheld smartphones to desktop computers. Data stored in the application is ready for use in patient management and downstream population-centered analyses and is securely stored on encrypted University of Michigan servers. MiDiET also features a triage algorithm that leverages objective cutoffs to determine whether any given patient requires care beyond the expertise of the MAs. The triage feature was built to accommodate MA-driven care providing ancillary support to local physicians. Use of MiDiET, however, was restricted to its data tabulation capabilities. The endocrinologists additionally produced education pamphlets for the MAs to distribute during consultations. Pamphlets were reviewed locally for content and appropriateness and then translated into Tamil to better serve the community in Trincomalee. All patients were consented prior to enlisting their participation in our pilot.

We identified and trained four medical assistants, each of whom had an affiliation with the Grace Girls’ Home (GGH). GGH is an orphanage and elder care facility that was built in Trincomalee in 2002. GGH offers food, shelter, healthcare, psychiatric attention, and a sense of community to all its residents. Two of the assistants are residents of GGH who were seeking employment; the other two are dedicated medical assistants who guide the care of the elders at GGH. Medical students and endocrinologists trained the MAs through both a hands-on, on-site approach as well as via video conferencing. Medical students provided ongoing support during implementation of the program by email, phone and weekly video conferencing. Regular discussions with local physicians were held to ensure that the needs of the hospitals were met by the program. Medical students and endocrinologists analyzed data that were entered into MiDiET by the trained MAs. Analysis was conducted through use of R-Studio, an open source software used to run R, a statistical programming language for assessing structured datasets. Descriptive data were summarized to evaluate the reach of the program.

### Impact

Implementation began in June 2016 and continued for one year. The MAs made weekly visits to the hospital’s diabetes clinic, where all patient encounters were documented. Descriptive data were collected on a cohort of patients (*n* = 93), documenting demographic information, fasting blood sugar, blood pressure and the medications being prescribed and taken (Fig. 1b). The medication lists were restricted to only those that were pertinent to the management of diabetes and cardiovascular disease. Only 14/93 (15%) of patients reported taking insulin. Every patient the MAs encountered received educational material on diabetes as well as nutritional guidelines specifically for people with diabetes. While return visits were encouraged, only 14/93 (15%) of the patients made at least one return visit within the 12-month study period. Of those patients, 3 out of 14 visited the clinic more than once. Through interviews with physicians, we learned that patient trust in Sri Lanka is highly linked to the physician, and trust for ancillary medical professionals is comparatively low, as there is a perceived disparity in the quality of care that one would receive from a physician versus a MA. Initial implementation at our partner hospitals has facilitated our acquisition of dedicated clinic space within the Selvanayakapuram Central Hospital, where we will further evaluate the efficacy, efficiency, and acceptance of our medical assistant program compared to usual care (involving primary care visits).

## Conclusions

Our implementation and maintenance of a medical assistant program in an under-resourced setting in Sri Lanka demonstrates that trained MAs can perform tasks that have medical utility. Existing resources in the context of an untapped labor force thus have the potential to increase capacity necessary for handling large patient volumes and reduce systemic congestion. While the MAs are currently compensated through our pilot, their competency produces a valid value proposition for government hospitals and/or private practices to buy in to a MA-driven service. Nurse case management has demonstrated improved glycemic control compared to primary care driven care in the United States [21]. If operated in an analogous manner, MA-driven care may achieve similar benefits. A formal study comparing usual care to MA-driven care, with clearly defined endpoints such as Hemoglobin A1C levels, blood pressure, and lipid management is thus an immediate future direction. Further studies evaluating the efficiency and acceptance of MA-driven care versus usual care will be essential to define the role MAs can play in care delivery. Furthermore, validating the accuracy and precision of data-entry as well as the technical acumen of the MAs will be important in ensuring that MA-driven care is of high quality.

While the pilot captured data from 93 patients from their first visits, the follow-up rate was quite low (14/93). Both the short duration of follow-up and negative perceptions of medical personnel who are not physicians may have contributed to the low number of follow up visits. The role of medical assistants is not widely recognized in Sri Lanka, similar to many other LMICs [13]. Enthusiastic physician endorsement of MAs will be critical to the future success of our model. Running parallel to negative public perception is the systemic deficit of proper diabetes education. The ministry of health’s official dietary guidelines (last published in 2011) places a large emphasis on the consumption of carbohydrate rich foods [22]. While these recommendations reflect cultural preferences regarding daily diets, high glycemic index foods are not traditionally recommended for people with diabetes. Official endorsement in the form of a nationally certified MA position that passes rigorous curricular standards can serve not only as a conduit to reduce systemic congestion and improve outcomes, but also as a public health measure to reduce incidence by cultivating a well-informed patient population. Our team has drawn up blueprints for a curriculum with a list of required competencies, inspired by equivalent counterparts in the United States. We aim to work with ministry officials to turn our pilot study into a nationally endorsed standard. Successful integration of our model into Sri Lanka’s health system will serve as an example that can be applied across the world in LMICs.

## Abbreviations

DM: Diabetes mellitus
EHR: Electronic health record
GGH: Grace Girls’ Home
LMIC: Low- and middle-income countries
MA: Medical assistant
MiDiET: Michigan Diabetes Evaluation Tool
